# Endocrine and Digestive Disorders Arising in Childhood in Down Syndrome and Their Cross-Talk

**DOI:** 10.3390/nu18121928

**Published:** 2026-06-14

**Authors:** Giuseppe Cannalire, Roberta Rotondo, Valentina Donini, Alessandra Fradusco, Marialaura Menzella, Anna Giuseppina Montani, Simone Pilloni, Tommaso Toschetti, Susanna Esposito, Giacomo Biasucci, Maria Elisabeth Street

**Affiliations:** 1 Pediatrics and Neonatology Unit, University of Parma, Guglielmo da Saliceto Hospital, 29121 Piacenza, Italy; g.cannalire@ausl.pc.it; 2 Department of Medicine and Surgery, University of Parma, 43121 Parma, Italy; roberta.rotondo@unipr.it (R.R.); annagiuseppina.montani@unipr.it (A.G.M.); susannamariaroberta.esposito@unipr.it (S.E.);; 3 Department of Translational Medical Science, Section of Pediatrics, University Federico II, 80131 Naples, Italy; 4 Pediatric Clinic, University Hospital of Parma, 43121 Parma, Italy

**Keywords:** Down syndrome, endocrine disorders, digestive disorders, autoimmunity, microbiota, dysbiosis

## Abstract

Down syndrome (DS), caused by trisomy 21, is associated with a wide spectrum of endocrine and gastrointestinal disorders that often arise early in life and significantly impact long-term health. This narrative review examines the pathophysiological mechanisms underlying these conditions, with a particular focus on their bidirectional interactions. Endocrine abnormalities in DS, including thyroid dysfunction, type 1 diabetes mellitus, growth impairment, and altered bone metabolism, occur at higher rates than in the general population and are largely driven by immune dysregulation, chronic inflammation, and gene dosage effects. Similarly, gastrointestinal disorders—ranging from congenital malformations to autoimmune conditions such as celiac disease—are highly prevalent and often present with atypical clinical features. Emerging evidence highlights the central role of gut dysbiosis, characterized by reduced microbial diversity and increased pro-inflammatory taxa, in modulating immune and metabolic pathways. This altered gut environment contributes to a chronic inflammatory state and may promote autoimmunity and endocrine dysfunction through the gut–endocrine–immune axis. Nutritional deficiencies and epigenetic factors, including microRNA dysregulation, further influence disease expression. Understanding this complex cross-talk is essential for improving clinical management. Integrated, multidisciplinary approaches and early screening strategies are crucial to optimize outcomes and guide future research in DS.

## 1. Introduction

Down syndrome (DS), caused by full or partial trisomy of chromosome 21, is the most common chromosomal abnormality in humans, with a prevalence of approximately 1:1000 live births worldwide [1,2]. The resulting gene dosage imbalance affects cellular homeostasis and organ development, leading to a broad and heterogeneous clinical phenotype. Individuals with DS typically present with characteristic craniofacial features, intellectual disability, growth delay, and an increased susceptibility to several comorbid conditions, including congenital heart defects, leukemia, Alzheimer’s disease, and endocrine dysfunctions, particularly involving the thyroid gland and autoimmune conditions [2].

Although the genetic basis of DS has been extensively characterized through genome-wide association studies of chromosome 21 and related regions [3,4], the molecular mechanisms linking trisomy 21 to systemic dysfunction remain incompletely understood.

Endocrine disorders are among the most frequent medical conditions in DS and often present early in life. Thyroid dysfunction, especially congenital or early-onset hypothyroidism, occurs at markedly higher rates (1:141 newborns) than in the general newborn population and reflects a multifactorial etiology involving thyroid dysgenesis, impaired hormone synthesis, immune dysregulation, and increased susceptibility to autoimmune thyroiditis. Altered hypothalamic–pituitary–thyroid axis set-points, resistance to thyroid-stimulating hormone, and inflammatory modulation of endocrine tissues have been proposed as contributing mechanisms [5,6]. Gastrointestinal and autoimmune disorders are similarly over-represented in DS, with celiac disease (CD) being one of the most prevalent autoimmune conditions in this population. Children with DS have a 6- to 10-fold increased risk of developing CD compared with the general population, likely due to impaired immune tolerance, increased intestinal permeability, and chronic mucosal inflammation [7,8,9]. The frequent coexistence of CD with autoimmune endocrine disorders, particularly T1DM and autoimmune thyroiditis, supports the concept of a shared pathogenic background characterized by aberrant T-cell activation and dysregulated Antigen presentation. Notably, gastrointestinal manifestations in DS derive from a bidirectional chemical communication between the gut’s endocrine network, and are often atypical or subtle, highlighting the importance of systematic screening to prevent long-term complications such as malabsorption, growth impairment, and micronutrient deficiencies [10,11].

Recent advances have expanded the understanding of DS beyond classical aneuploidy toward an integrated pathophysiological model in which gene dosage effects, chronic inflammation, epigenetic regulation, metabolic alterations, micronutrient imbalance, and gut dysbiosis converge to shape clinical variability and disease susceptibility.

A key contributor to the DS phenotype is the overexpression of dosage-sensitive genes and regulatory elements located on chromosome 21, as several microRNAs (miRNAs) have emerged as critical epigenetic amplifiers of gene dosage effects in DS and collectively affect immune signaling, oxidative balance, neurodevelopment, endocrine axes, and gastrointestinal integrity [12,13,14,15,16]. In addition, individuals with DS exhibit a distinctive immune profile marked by thymic hypoplasia, reduced T- and B-cell maturation, impaired regulatory T-cell function, and constitutive activation of interferon pathways [17,18]. This immune dysregulation is reflected in a chronic low-grade inflammatory state that contributes to heightened susceptibility to autoimmune diseases [19,20].

Finally, emerging evidence suggests that DS is associated with specific gut dysbiosis, marked by reduced microbial diversity, enrichment of pro-inflammatory taxa, and depletion of butyrate-producing bacteria. This altered microbiota may interact with the pre-existing immune and metabolic imbalance to promote chronic inflammation, autoimmunity, impaired nutrient absorption, and endocrine dysfunction, highlighting the relevance of the gut–immune–metabolic axis and of the endocrine and digestive cross-talk in DS pathophysiology. Although current data are limited by small sample sizes and methodological heterogeneity, consistent findings across studies support the biological relevance of microbiota alterations and underscore the need for longitudinal, standardized investigations to explore microbiota-targeted interventions, including dietary strategies and probiotics, as potential tools to improve clinical outcomes in individuals with DS [21].

The aim of this narrative review is to provide a comprehensive overview of endocrine and gastrointestinal disorders in DS, with a particular focus on the pathophysiological mechanisms and their potential cross-talk to provide useful information for further understanding and research. Moreover, we have focused on the current evidence available on the effects of nutritional supplementation on these conditions in DS.

## 2. Materials and Methods

This narrative review was conducted to provide a comprehensive overview of endocrine and gastrointestinal disorders in children and adolescents with Down syndrome (DS), with particular emphasis on their pathophysiological interactions and the potential role of the gut–endocrine–immune axis. To identify the most relevant evidence, a structured literature search was performed across PubMed/MEDLINE, EMBASE, Scopus, ScienceDirect, and Google Scholar. Publications from 1975 to 2025 were considered, and the last search was conducted in December 2025.

Eligible publications included systematic reviews, meta-analyses, narrative reviews, randomized controlled trials, controlled clinical trials, observational studies, case series and case reports, guidelines, consensus statements, and recommendations from scientific societies. Studies were considered relevant if they focused on pediatric patients (0–18 years) with Down syndrome and addressed endocrine disorders (including thyroid dysfunction, autoimmune thyroid disease, type 1 diabetes mellitus, growth disorders, adrenal and parathyroid disorders, and pubertal abnormalities), gastrointestinal disorders (including celiac disease, inflammatory bowel disease, intestinal inflammation, malabsorption, and microbiota alterations), or the potential interactions between these conditions.

The following combinations of keywords were used to obtain the search strings: (Down syndrome OR trisomy 21 OR DS) AND (newborn* OR neonat* OR infan* OR toddler* OR child* OR children OR adolescent* OR pediatr* OR paediatr* OR youth* OR teen* OR baby OR babies) AND (endocrinopath* OR endocrine disorder* OR thyroid dysfunction* OR hypothyroidism OR hyperthyroidism OR Hashimoto* OR Graves* OR diabetes mellitus OR type 1 diabetes OR T1DM OR autoimmune endocrinopath* OR hypoparathyroidism OR adrenal insufficienc* OR adrenal insufficiency OR glucocorticoids OR mineralcorticoids OR growth hormone deficienc* OR GH deficienc* OR short stature OR delayed puberty OR precocious puberty OR pubertal disorder*) AND (enteropathies* OR gastrointestinal disorder* OR gastrointestinal disease* OR coeliac disease OR celiac disease OR gluten-sensitive enteropathy OR inflammatory bowel disease OR IBD OR Crohn* OR ulcerative colitis OR intestinal inflammation OR chronic diarrhea OR malabsorption OR leaky gut OR gut inflammation OR intestinal permeability OR microbiota OR gut microbiome OR intestinal microbiome OR dysbiosis OR microbial composition OR gut flora OR gut bacteria OR intestinal bacteria) AND (cross-talk OR crosstalk OR interaction* OR axis OR link* OR association OR relationship OR communication OR signaling OR correlation OR network OR gut–brain axis OR gut-endocrine axis OR gut-immune axis OR immune-microbiota interaction* OR microbiota-endocrine interaction* OR endocrine-gut axis OR autoimmunity OR prebiotic* OR probiotic* OR pre- and probiotics OR postbiotics).

In addition, manual searches of reference lists of selected articles were performed to identify further relevant publications. The search was restricted to English-language full-text articles. Following duplicate removal, titles and abstracts were screened for relevance to the review topic, and potentially eligible articles were assessed for inclusion according to the predefined thematic criteria. The selected studies were then analyzed and synthesized narratively to provide an integrated overview of endocrinopathies, gastrointestinal disorders, microbiota alterations, and autoimmune conditions in Down syndrome, with particular emphasis on their reciprocal interactions and the potential role of nutritional interventions.

Given the narrative nature of this review, the literature search was intended to ensure broad and comprehensive coverage of the available evidence rather than to perform a formal systematic review. Therefore, no PRISMA-guided study selection process, formal risk-of-bias assessment, or quantitative synthesis was undertaken.

## 3. Results

### 3.1. Inflammation, Immunity, and Epigenetics

Among genetic syndromes, DS is the one most commonly linked to immune dysregulation [22]. People with DS have significantly higher rates of autoimmune conditions, including autoimmune thyroid disease, type 1 diabetes, and CD, occurring roughly four to six times more often than in the general population [22]. Rheumatologic disorders such as arthritis and systemic lupus erythematosus, dermatologic conditions including alopecia areata and vitiligo, and neurological diseases like Moyamoya have also been observed to be increased [19,22]. Table 1 summarizes the most common autoimmune conditions presenting in individuals with DS.

Multiple alterations, affecting components of both the innate and adaptive immune responses, have been suggested to account for the increased predisposition of individuals with DS to autoimmune diseases.

Human leukocyte Antigen (HLA) subtypes also play a complex role in individuals with DS. A study by Nicholson et al. in 1994 showed that the class II major histocompatibility complex (MHC II) allele DQA10301, regulated by immune-related genes on chromosome 21, was strongly over-represented in individuals with DS and autoimmune hypothyroidism [23]. A similar positive association was reported by Book et al., who showed that seven of eight children with DS and CD carried the high-risk HLA genotype DQA10501–DQB1*0201, compared with 13 of 80 children with celiac disease alone, suggesting that this high-risk HLA genotype is more common in children with both DS and CD than in those with CD alone [24].

Individuals with DS exhibit significant B-cell abnormalities, including reduced switched memory B cells and an increased frequency of autoreactive CD21^low B cells, along with a distinctive immunoglobulin profile characterized by initially elevated IgG levels, generally normal IgA, and reduced IgM and IgE levels [18]. T-cell defects are evident early in life, already at the thymic level, with reduced naïve T cells, accelerated thymocyte maturation, and impaired memory T-cell expansion, collectively contributing to premature immune senescence [25]. Expression of the AIRE gene, which is essential for negative selection of autoreactive T lymphocytes, is often reduced, thereby increasing susceptibility to autoimmunity [26]. CD8^+^ and CD4^+^ T cells display increased production of pro-autoimmune cytokines, while regulatory T cells (Tregs) appear functionally intact, but effector T cells exhibit resistance to Treg-mediated suppression [27]. Dysregulation of innate immunity further includes overexpression of Toll-like receptors, particularly TLR-2, and functional defects in neutrophils and natural killer cells, promoting both recurrent infections and autoimmune susceptibility [18].

Moreover, in individuals with DS, trisomy of chromosome 21 leads to overexpression of the interferon receptor genes, resulting in increased basal interferon activity [28,29] which further enhances susceptibility to autoimmune diseases such as Hashimoto’s thyroiditis, celiac disease, type 1 diabetes, and dermatological conditions.

Epigenetic changes have also been reported in connection with chromosome 21 trisomy. MiRNAs, small endogenous non-coding RNAs that act as negative regulators of target gene expression, encoded on chromosome 21, including miR-155, miRNA-125b, miRNA-99a, and miRNA-802, are consistently overexpressed and play central roles in immune regulation, thyroid autoimmunity, insulin signaling, oxidative stress responses, and intestinal epithelial homeostasis [30,31,32]. Additional differentially expressed miRNAs, such as miRNA-1246, miRNA-138-5p, miRNA-30c, miRNA-145, and miRNA-183, have been implicated in p53-mediated pathways, neurodevelopment, stress responses, and immune regulation through integrated network analyses [33,34,35]. Functional enrichment studies showed that the miRNA-targeted genes involved played a role in protein folding, chromatin organization, lipid metabolism, and oxidative stress, reinforcing the role of miRNAs as key regulators linking trisomy 21 to multisystem pathology [35]. Increasing studies suggest the importance of miRNAs in the regulation of the growth plate and growth hormone (GH)-insulin-like growth factor (IGF) axis, and some of the dysregulated miRNAs in DS are involved in the regulation of these pathways and likely contribute to impaired linear growth [36,37]. Specific miRNA dysregulation could represent a molecular link binding the inflammatory component underlying DS to growth impairment and insulin resistance [37].

Furthermore, the overexpression of miRNAs encoded on chromosome 21, such as miRNA-99a, let-7c, miRNA-125b-2, miRNA-155, and miRNA-802, leads to the downregulation of key innate immune regulatory and anti-inflammatory genes [27]. These include, for example, complement factor H and specific Toll-like receptor (TLR) miRNAs, both of which are critical in the initiation of inflammation and autoimmunity [22]. A recent study through in vivo and in vitro analyses showed that miRNA-125 and miRNA-155 are overexpressed in B cells from individuals with DS and would be potential contributors to the well-recognized immunodeficiency that characterizes this syndrome [31].

**Figure 1:**
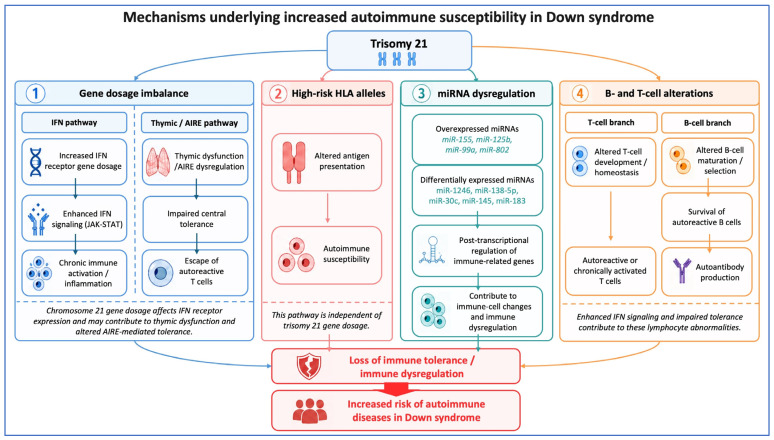
Mechanisms underlying autoimmune susceptibility in DS. DS is associated with an increased risk of autoimmune diseases through the combined effects of genetic predisposition (gene dosage imbalance and high-risk HLA alleles), epigenetic alterations (altered microRNA expression), and immune-cell dysfunction. These factors lead to enhanced interferon signaling, promote chronic inflammation, reduce immune tolerance, and favor autoantibody production, ultimately increasing susceptibility to autoimmune conditions such as autoimmune thyroid disease, CD, and type 1 diabetes. Genetic factors and epigenetic alterations, including specific miRNA expression, contribute to the increased risk of autoimmune diseases in patients with Down syndrome. Abbreviations: IFN (interferon); HLA (Human Leukocyte Antigen); miRNA (microRNA); Treg (regulatory T cell).

Taken together, these epigenetic/genetic factors also likely help explain the high prevalence of autoimmune disorders in this population. Overall, the interaction between gene overexpression, immune dysregulation, and chronic inflammation provides a mechanistic link between the DS genotype and the complex range of autoimmune phenotypes observed in this population. The mechanisms underlying the increased risk of autoimmune disease in patients with DS are summarized in Figure 1.

In addition to the genetic and immunological abnormalities described above, increasing attention has been directed toward the potential contribution of the gut microbiota to immune dysfunction in individuals with DS. The intestinal microbiota plays a key role in maintaining immune homeostasis through the production of metabolites such as short-chain fatty acids (SCFAs) and tryptophan-derived compounds, which support epithelial barrier function, promote regulatory T-cell differentiation, and contribute to the control of inflammatory responses. Alterations in microbial composition may disrupt these protective mechanisms, resulting in reduced production of immunoregulatory metabolites and increased intestinal permeability. The following translocation of microbial products can activate innate immune pathways, including Toll-like receptor signaling, leading to enhanced secretion of pro-inflammatory cytokines and a shift toward Th1- and Th17-mediated immune responses. Concerning DS, where interferon signaling, lymphocyte function, and immune regulation are already deeply altered, microbiota-derived inflammatory signals may further amplify chronic immune activation and cause the loss of self-tolerance. Although the causal relationship between dysbiosis and autoimmunity in DS remains to be fully developed, current evidence suggests that alterations in the gut microbiota may contribute to the inflammatory environment that characterizes the syndrome, and may represent an additional factor involved in the development of autoimmune manifestations [38].

### 3.2. Endocrine Disorders

Individuals with DS are predisposed to the development of endocrine disorders. A recent literature review has shown that endocrine and metabolic disorders, including thyroid dysfunction, T1DM, obesity, osteopenia/osteoporosis, and growth abnormalities, are significantly more frequent in these patients compared with the general population [39].

#### 3.2.1. Thyroid Disorders

It is widely acknowledged that individuals with DS are at higher risk of thyroid hormone derangements compared with the general population. These abnormalities encompass the full spectrum of thyroid disorders, including congenital hypothyroidism, acquired subclinical hypothyroidism, acquired overt hypothyroidism, and hyperthyroidism [40]. The incidence of these disorders in pediatric patients with DS ranges from 12% to 27%, whereas in the general pediatric population it is 2–5% [41].

In contrast to the general population, in individuals with DS, Hashimoto thyroiditis generally presents earlier, most commonly with subclinical hypothyroidism. In addition, this population exhibits a higher susceptibility to conversion to Graves’ disease. No gender-related pattern for Hashimoto thyroiditis is reported in DS [42].

The thyroid disorders in DS are strongly linked to immune dysregulation, driven by thymic hypoplasia, altered T-cell maturation, and heightened by increased pro-inflammatory cytokine activity [42].

Subclinical hypothyroidism is also ascribed to a delay in the maturation of the hypothalamic–pituitary–thyroid axis and is thought to sometimes be a transient and self-limiting process that does not require treatment. Different hypotheses have been put forward, including a cutdown of the dopaminergic tone both in the hypothalamus and pituitary gland, which would lead to increased TSH secretion. This, in turn, would lead to TSH receptor downregulation, restoring normal baseline values of thyroid hormones. Some degree of receptor resistance has also been hypothesized [5].

Moreover, the increased chronic oxidative stress due to overexpression of redox-related genes would increase cellular vulnerability, including thyrocytes. Selenium deficiency and impaired antioxidant enzyme activity further compromise thyroid protection, collectively predisposing individuals with DS to thyroid dysfunction [43]. Moreover, epigenetic modifications, such as aberrant methylation of thyroid-related genes, would further impair thyroid function in DS [5].

Clinical assessment of thyroid disease in DS is challenging because systemic manifestations of hypothyroidism overlap with typical trisomy-21 features (hypotonia, dry skin, slower mental development, increased irritability, and behavioral disturbances), thus requiring the need for regular screening. Graves’ disease in children with DS is symptomatic and easy to diagnose, often associated with other autoimmune diseases [11,44]. A series of 28 children and adolescents [45] were evaluated, and clinical characteristics were reported at an earlier age at presentation, with no gender predominance and a prior higher frequency of Hashimoto thyroiditis. A further study highlighted difficulty in controlling hyperthyroidism with medical treatment (Carbimazole) [46].

#### 3.2.2. Growth Disorders

Short stature is a key feature of DS. Children with DS exhibit a distinct growth pattern, characterized by shorter stature, typically approximately two standard deviations below the mean with respect to the general population, and reduced growth velocity. Trisomy 21-specific growth charts have been developed to monitor height and weight in this population. Growth assessment should be performed using these condition-specific charts as they are essential for detecting deviations in growth velocity that may reflect underlying medical conditions [47].

Growth retardation in children with DS begins prenatally. After birth, growth velocity is most markedly reduced by 6 months and 3 years of age, and puberty is characterized by an attenuated pubertal growth spurt [48]. Short stature is primarily attributable to reduced length of limbs, while the trunk size is generally preserved. Currently, different explanations can be put forward to understand these changes, as previously mentioned. In DS, the GHRH–GH–IGF-1 axis is recognized to be dysregulated, primarily due to hypothalamic dysfunction with preserved pituitary responsiveness. Circulating growth hormone (GH) levels are generally not overtly deficient; however, insulin-like growth factor 1 (IGF-1), the main mediator of GH action, is consistently reduced in children with DS, particularly in early childhood, and remains low throughout life. Reduced IGF-1 levels likely reflect the combined effects of genetic factors, nutritional status, oxidative stress, and chronic low-grade inflammation. Specifically, elevated pro-inflammatory cytokines (TNF-α, IFN-γ, and IL-6) reduce hepatic IGF-1 production by causing GH resistance [49]. Furthermore, triplication of chromosome 21 genes leads to elevated levels of pro-inflammatory cytokines and slows the rate of endochondral bone growth, decreasing the expression of aggrecan and collagen in the cartilage matrix [49]. In addition, miRNA dysregulation has negative effects on key pathways involved in the growth processes, as described above [36,37].

Some studies highlighted that GH treatment in children with DS has a beneficial short-term effect on linear growth as it increases growth velocity compared with untreated DS children [50]. However, in most studies, this benefit disappears after discontinuation of therapy, and there are insufficient data on long-term effects of treatment through to final height. Although concerns have been raised regarding a potential increased risk of leukemia, a condition for which individuals with DS already have a higher baseline risk, no evidence of an association between GH therapy and leukemia has emerged from studies published to date [50].

#### 3.2.3. Weight-Related Disorders

Weight-related disorders in individuals with DS represent a complex and multifactorial issue, encompassing both undernutrition and overweight. Infants with trisomy 21 typically present with lower birth weight compared with children without chromosomal abnormalities; however, during adolescence and adulthood, the presence of multiple comorbidities and intrinsic features of the syndrome predispose these individuals to excessive body weight [51].

Children with DS present with poor weight gain from birth to infancy, mostly because of feeding difficulties due to hypotonia and macroglossia or associated comorbidities, including heart defects and gastrointestinal malformations [52]. The increased prevalence of overweight and obesity in this population is likely attributable to multiple factors, including reduced basal metabolic rate, low levels of leptin, hypotonia, heightened susceptibility to systemic inflammation, and the frequent coexistence of hypothyroidism. Additionally, impaired mobility and motor coordination may limit physical activity, thereby promoting a predominantly sedentary lifestyle. Moreover, several studies have linked telomere shortening, associated with accelerated aging in DS, to increased body mass index (BMI) and adiposity. In DS, obesity may worsen other conditions such as obstructive sleep apnea and cardiovascular disease [53]. Furthermore, it must be considered that weight gain leads to a reduction in physical activity, creating a vicious circle that favors the onset of true obesity, which aggravates, especially in the developmental age, some pathologies such as orthopedic conditions.

Obesity in DS is also associated with a high prevalence of pre-diabetes and diabetes, with high levels of insulin and hypertension, presenting mostly in adult age [54]. Indeed, people with DS have a higher risk of developing type 2 diabetes, particularly at a younger age (5–14 years) compared to the general population. Patients with DS also present an increased rate of non-alcoholic fatty liver disease, which is closely associated with insulin resistance [55].

Besides obesity, genetic predisposition in DS plays a role in this. In fact, a number of genes located on the DS critical region of chromosome 21, including dual-specificity tyrosine phosphorylation regulated kinase 1A (DYRK1A) and regulator of calcineurin 1 (RCAN1), are thought to contribute to diabetes. For instance, Wang et al. explored the role of DYRK1A as a regulator of human β-cell proliferation and found that overexpression of DYRK1A attenuated β-cell proliferation in human islet cells, and conversely, reduced endogenous DYRK1A led to an increase in human β-cell proliferation [56].

Systemic hypertension is uncommon in DS, with a lower risk of developing hypertension even with higher rates of obesity and sedentary behavior. If present, it is usually secondary to other conditions such as thyroid disease (hyper/hypothyroidism), kidney disease, or medication side effects. Genetic factors (e.g., RCAN1, DYRK1A on chromosome 21) are thought to offer protection against high blood pressure [54].

#### 3.2.4. Type 1 Diabetes Mellitus (T1DM)

T1DM is an autoimmune condition caused by immune-mediated destruction of pancreatic β-cells. Individuals with DS exhibit a higher prevalence of diabetes mellitus than the general population. Some evidence shows that both type 1 and type 2 diabetes occur at approximately a threefold higher rate in individuals with DS compared with the normal population [57]. One study reported a four times higher prevalence of T1DM in DS children and adolescents than in the general population (0.7% vs. 0.17%) [58].

A further study on DS children reported a very early presentation of T1DM: about 17% are diagnosed by an age of 2 years compared with 0.4% in the general population. Clinical presentation in individuals with or without DS is similar. Notably, despite earlier onset, individuals with DS and T1D often achieve better metabolic control with lower insulin doses, possibly due to structured routines or lifestyle [19].

These findings may be in part ascribable to the increased prevalence of diabetes-associated HLA class II genotypes in children with both DS and T1DM compared with healthy controls; however, children with DS and T1DM are less likely to carry the highest-risk HLA genotypes (i.e., DR4-DQ8/DR3-DQ2) and instead more frequently harbor lower-risk alleles. This observation has led to the hypothesis that additional susceptibility factors, potentially involving genes located on chromosome 21, may contribute to the increased penetrance of T1DM in children with DS. For instance, the coexistence of increased gene dosage and point mutations affecting both the AIRE gene (21q22.3) and its promoter region may result in overexpression of its transcript, promoting the production of autoantibodies against pancreatic islet cells [38]. The chronic low-grade inflammation with overexpression of pro-inflammatory cytokines in individuals with DS, largely attributable to a constitutional dysregulation of the interferon and interleukin-6 signaling pathway, may also play a role [19,20]. This facilitates the development of autoimmune processes [19,20].

#### 3.2.5. Gonadal Function and Puberty Disorders

Apparently, children and adolescents with DS have a high incidence of abnormalities in sexual development, but research in this population is scarce. In females, the described abnormalities include hypogonadism; in males, the reported defects include ambiguous genitalia cryptorchidism, micropenis, small testes, low sperm count, and scant axillary and facial hair [59].

Previous studies have evidenced that male patients with trisomy 21 may exhibit hypergonadotropic hypogonadism, characterized by increased FSH and LH levels, detectable as early as infancy and progressing from late puberty into adulthood. This condition is probably due to dysfunction of both Leydig and Sertoli cells [6,60].

Furthermore, several studies have shown that male individuals with DS have lower total testosterone concentrations compared with control populations, a finding that may be attributable to Leydig cell impairment [61].

Concerning pubertal development, a recent study from Turkey compared age at pubertal onset, age at menarche, and time to attainment of Tanner stage V in 51 pubertal female patients with DS [62]. These authors observed that the onset of thelarche occurred significantly later in girls with DS compared with their healthy peers (10.4 yr vs. 9.65 yr). Likewise, the age at attainment of Tanner stage V breast development was delayed in girls with trisomy 21 (15 yr vs. 14.2 yr). In contrast, menarche appeared earlier in girls with DS compared with healthy controls. Although the age at pubertal onset in boys with DS did not differ from that of healthy children, the attainment of the final pubertal stage appeared to occur later than in typically developing peers [62]. Nevertheless, studies including larger patient cohorts are needed to more accurately characterize the pubertal trajectory in children with DS and their causes.

#### 3.2.6. Bone Metabolism Disorders

Bone mass accrual during childhood and adolescence is a critical determinant of skeletal health in adulthood, as osteopenia in childhood may manifest as osteopenia and osteoporosis in later adult life. Individuals with DS consistently present reduced bone mass compared with both peers without intellectual disability and individuals with intellectual disability without trisomy 21 [63].

The prevalence of osteoporosis is reported to be greater than 50% in both adult male and female patients with DS. Furthermore, peak bone mass is attained earlier (around age 20–25 yr) and declines faster thereafter, suggesting an accelerated aging on the skeleton [64].

The etiology of osteoporosis in DS is recognized as multifactorial. Factors such as muscle hypotonia, reduced physical activity, insufficient calcium and vitamin D intake, hypogonadism, growth retardation, and thyroid dysfunction may collectively impair skeletal maturation and bone mass accrual, thereby increasing susceptibility to bone fragility and fractures. Notably, accumulating evidence suggests that DS represents a genetic condition intrinsically associated with compromised bone health [65].

DS children and adolescents present a markedly high prevalence of vitamin D deficiency as they typically spend less time outdoors and engage in lower levels of physical activity [63]. Elevated parathyroid hormone levels are associated with 25(OH)D deficiency that removes calcium from bones, underscoring the critical role of adequate vitamin D status in maintaining normal bone metabolism [66].

Regular monitoring of bone health and vitamin D levels is advisable due to higher prevalence of comorbidities affecting bone metabolism [67,68,69].

### 3.3. Gastro-Intestinal Disorders

A number of organic and functional conditions have been described in DS. We first present commonly reported anatomical malformations and functional changes, then specific disorders in the paragraphs below. Interactions and signaling mechanisms between endocrine and gastro-intestinal disorders play a crucial role in maintaining metabolic homeostasis and overall health.

#### 3.3.1. Anatomical Malformations and Functional Disorders

Gastrointestinal (GI) malformations are described in 4 to 10% of patients with DS [70]. Among the hypotheses that try to explain the association between gastrointestinal alterations and DS is the idea that changes in neurotropic factors occurring during the embryogenesis of the central and enteric nervous systems probably play a role [71].

Among the structural anomalies of the GI tract, duodenal atresia (DA) is the most commonly reported. In addition, 25% of infants born with DA also have a diagnosis of DS [72]. Anal stenosis and/or atresia have been documented in 1–4% of infants with DS [47]. Esophageal atresia and tracheo-esophageal fistulas are less common (0.3–0.8% of cases), but still more frequent than in the general population [73].

In addition, imperforate anus, annular pancreas, congenital megacolon, esophageal atresia, esophageal compression by anomalous subclavian, and congenital duodenal membrane have been reported [70].

Hirschsprung’s disease is also reported in DS individuals. This disorder has an estimated prevalence of 1–3% in the DS population, and about 5% of patients with Hirschsprung’s disease have DS [74]. Literature reports that DS individuals have a worse prognosis since the surgical removal of the aganglionic segment in these patients does not tend to resolve bowel dysfunction [47].

Among functional GI disorders associated with DS, constipation is frequent because of several contributing factors, including hypotonia, poor diet, and reduced physical activity. In addition, gastroesophageal reflux and feeding difficulties are typically present, possibly due to disorders in oral motor function and related to neurological dysfunction of the esophagus [47,70].

A recent study evaluated the prevalence of GI disorders during a 10-year follow-up period in a public referral outpatient clinic for people with DS [70]. The study included more than 1200 patients. Among these, 612 (about 50%) presented with GI disorders. The most prevalent disorder was constipation (49%), and gastroesophageal reflux disease was also quite commonly observed (14% of cases), associated with general hypotonia involving esophageal muscles, especially the lower esophageal sphincter [70].

#### 3.3.2. Celiac Disease

Celiac disease (CD) deserves special mention as several studies have investigated its association with DS.

The mechanism that poses DS individuals at increased incidence of CD is not yet fully understood. The data in the literature suggest an association with increased interferon-α receptor expression coded on chromosome 21. As previously described, the augmented incidence of CD is also influenced by immune alterations intrinsic to trisomy 21, including impaired central and peripheral immune tolerance and dysregulated cytokine signaling. Moreover, since interferon-α plays a role in inducing intestinal immune responses, the potential increase in receptors observed in DS may trigger the autoimmune response triggering CD [75].

The first case of a patient with DS and CD published in the literature was described in 1975 [76]. The rate of CD among children with DS is estimated to be between 1 and 5%, according to the American Academy of Pediatrics (AAP) Health Supervision Guidelines for Children with Down Syndrome published in 2022 [77].

Studies from the literature around the world show variable data concerning the prevalence of CD in children with DS, with values ranging from 0% to 19% [7]. The risk of CD in children with DS increases with increasing age, ranging from a fourfold increase during the first year of life to an almost tenfold increase in children with DS aged 5–10 years. This may be related to the fact that small children with DS may have difficulties in communicating CD-related symptoms, resulting in an underdiagnosis of CD in younger children with DS. A further study from Colombia, carried out between 2014 and 2018 [78], reported a higher histopathology-confirmed CD frequency in DS children compared with children without DS (3.1% vs. 1.8%, *p* = 0.432), even though the results were not statistically significant. A further study [79] from two major hospitals in Abu Dhabi assessed the prevalence of CD, type 1 diabetes mellitus, and hypothyroidism in DS. The prevalence of CD was estimated through IgA anti-transglutaminase positivity. Of 92 patients considered, only one (1.1%) had CD with high levels of anti-tTG IgA antibodies (36 U/mL). The authors concluded that in the United Arab Emirates, there was no link between DS and celiac disease, even though the numbers of the studied patients were actually too small to draw definite conclusions and total IgA levels were not measured, thereby potentially excluding children with IgA deficiency from the diagnosis of CD [79]. Among the reasons that may explain the differences in the prevalence of CD among DS individuals, different genetic predisposition, diets, and other environmental factors around the world must be taken into consideration [9].

The clinical presentation of CD has been reported to be quite variable, with prevailing GI symptoms among patients with active CD including abdominal pain, constipation, abdominal distension, poor weight gain, flatulence, diarrhea, and vomiting [80]. CD may present with atypical symptoms as menstrual disturbances [81].

Indications on screening for CD in children with DS are still not conclusive, ranging from universal screening to screening limited to individuals with suggestive symptoms [77]. Another approach, as described by Wouters et al. [82], could be to target routine serological screening in children with DS and HLA DQ2/HLA DQ8 alleles.

#### 3.3.3. Inflammatory Bowel Diseases (IBDs)

Data concerning the risk of IBD in individuals with DS are still not conclusive. The prevalence of ulcerative colitis and Crohn’s disease in DS individuals seems to be increased compared to the general population [83], although in some studies the association between these conditions was not evident [84]. While the direct link to inflammatory bowel disease is still under investigation, chronic dysbiosis and immune alterations place DS patients at a high risk of chronic intestinal inflammation [2,84,85]. Higher amounts of pro-inflammatory cytokines (including IL-1α, IL-1β, IL-6, and TNF-α) and lower numbers of anti-inflammatory cytokines (including G-CSF and angiogenin) in individuals with DS compared to non-DS volunteers can modify the intestinal environment and lead to the proliferation and colonization of opportunistic pathogens, invasion of the body’s immune system, and enhance inflammation [41]. The impact of the extra copy of four out of the six IFN-receptor genes in DS individuals and its possible association with increased risk of IFN-related diseases in these individuals is of interest but requires deeper analysis and further studies [86].

#### 3.3.4. Hepatitic Disease

Hepatic disorders such as steatosis, biliary lithiasis, and primary sclerosing cholangitis are rarely observed in DS [70].

The literature reports sporadic isolated cases of autoimmune hepatobiliary diseases, including autoimmune hepatits and primary sclerosing cholangitis, in association with other autoimmune conditions (e.g., Hashimoto thyroiditis, Graves’ disease and alopecia areata) [87]. Since the symptoms of these diseases (fatigue, weight loss …) do not usually suggest a clear diagnosis, they must be searched and strictly monitored in people with DS [88]. One case series of seven DS patients reported a presentation of autoimmune hepatitis at a median age of 10 years (range 3–15 years). All seven patients were Antinuclear antibodies (ANA)+ and Smooth Muscle antibodies (SMA)+ while none were anti-Liver-Kidney Microsomial 1 ant59ibodies (LKM1)+.

### 3.4. Gut Dysbiosis and Metabolomic Changes

Recent evidence suggests that the systemic health of individuals with DS is profoundly influenced by the intestinal environment. DS is consistently associated with significant intestinal dysbiosis, characterized by reduced bacterial diversity and an expansion of pro-inflammatory bacterial taxa [89].

These alterations are consistent with the broader immune dysregulation and chronic inflammatory state described in DS, suggesting a bidirectional interaction between gut microbiota and host immunity [18,90]. A key feature of this dysbiotic state is the reduction in short-chain fatty acid (SCFA)-producing bacteria, particularly butyrate-producing genera, which play a crucial role in maintaining intestinal epithelial integrity, regulating tight junction function, and modulating immune responses. SCFAs are also involved in anti-inflammatory signaling pathways; therefore, their depletion may contribute to a pro-inflammatory milieu. In parallel, the formation of dysbiotic Biofilms may further exacerbate mucosal inflammation [91], contributing to increased intestinal permeability (“leaky gut”). This condition facilitates the translocation of luminal Antigens and microbial products into systemic circulation, promoting immune activation and sustaining chronic inflammation. Such mechanisms may contribute to the increased susceptibility to immune-mediated enteropathies observed in DS, including protein-losing enteropathy and villous atrophy [92].

Recent studies have indeed documented distinctive microbiota profiles in DS. In detail, intestinal microbiota is enriched with a number of pro-inflammatory bacteria (ex. Prevotella, Escherichia/Shigella) alongside an altered fecal metabolome [21]. These microbial shifts are correlated with elevated circulating cytokine levels including IL-1α, IL-1β, IL-6, and TNF-α, supporting the presence of a chronic low-grade inflammatory state linked to gut dysbiosis [21,90].

In addition to compositional changes, metabolomic analyses have revealed significant alterations in metabolic pathways related to amino acid metabolism and energy homeostasis. In particular, disruptions in branched-chain amino acids, ketone body metabolism, and SCFA-related pathways have been described [21,85]. These metabolic shifts may influence multiple biological processes, including immune cell function, oxidative stress responses, and endocrine signaling, thereby contributing to the multisystemic phenotype of DS.

Finally, gut dysbiosis may also impair nutrient metabolism and absorption, including the bioavailability of key micronutrients such as Folate (vitamin B9) and cobalamin (vitamin B12). These alterations may further impact neurodevelopmental and metabolic pathways, potentially contributing to cognitive dysfunction and metabolic abnormalities in individuals with DS [93]. Moreover, gut microbiota-derived metabolites may contribute to gut–brain axis alterations, potentially influencing neuroinflammatory processes and cognitive function in DS [85,94].

### 3.5. Gut–Endocrine Axes Cross-Talk

Overall, DS individuals present a dysregulation of the integrated “gut–endocrine–immune” system with subsequent endocrine and gastrointestinal disorders (Table 2). Trisomy 21 is now understood to intrinsically alter the immune system [19], which likely explains why organ-specific autoimmune diseases (thyroiditis, CD, T1DM, etc.) cluster in DS [95]. Notably, these conditions often co-occur in the same individual, essentially representing a partial polyautoimmune syndrome [96].

Such observations have prompted routine screening recommendations in DS care: thyroid function is checked at least annually throughout life, and celiac antibodies are periodically evaluated (with some guidelines advising universal celiac screening by age 2–3 years) [95]. Early identification of these comorbidities is critical, as untreated celiac disease or thyroid dysfunction would exacerbate developmental and metabolic issues in DS.

Importantly, emerging evidence includes alterations in the gut microbota in the pathogenesis of autoimmunity and endocrine dysfunction in DS. Therefore, dietary habits and intestinal dysbiosis will further modulate the immune system toward inflammation. Children and adults with DS frequently have poor diet quality, low activity levels, and a propensity to develop obesity—factors which can skew the gut microbiota composition and promote further systemic inflammation [95]. Notably, transplantation of DS-associated gut bacteria into mouse models has been shown to induce features including neurobehavioral changes, highlighting the far-reaching influence of the DS gut microbiome on host physiology. A reported case study showed that a child with DS tolerated fecal microbiota transplantation well, resulting in a more stable and diverse gut microbiome after the procedure [97]. Although research on the gut–endocrine cross-talk in DS is recent, these findings suggest that an “unhealthy” microbiome (potentially stemming from nutritional factors) does contribute to autoimmune endocrine triggers in DS [95,98].

Indeed, adequate levels of micronutrients such as vitamin D, often deficient in individuals with DS, are needed for immune regulation, and lower vitamin D status in DS has been linked with sedentariness and poor diet [95]. Such nutrient deficiencies may further skew immune homeostasis and thyroid function, compounding the risk of autoimmunity [19,95].

Overall, gastrointestinal disease was associated with an increased risk of endocrine disease, and conversely, endocrine disease also increased the risk of gastrointestinal diseases. For example, chronic constipation is a common problem in DS that can have multiple etiologies; thus, a DS patient presenting with constipation may warrant evaluation for both thyroid hormone levels and celiac serology, since these treatable endocrine/gut conditions often underlie the symptom. Conversely, endocrine abnormalities can adversely affect gastrointestinal and metabolic status. Hypothyroidism, in particular, has cascading effects on the gut. Low thyroid hormone levels reduce gut motility (worsening constipation) and can alter the gut microbiota composition, as thyroid hormone is known to influence bile acid metabolism and intestinal transit time, and can lead to excessive weight gain and hyperlipidemia, further disturbing metabolic homeostasis. Likewise, CD in DS exemplifies gut pathology, affecting endocrine and nutritional status. As DS patients often have subclinical or atypical celiac presentations, the condition may quietly impair nutrient absorption (causing iron-deficiency anemia, low vitamin D, etc.) and contribute to poor growth before it is recognized [66]. Many “classic” celiac symptoms, such as short stature, developmental regressions, behavioral disturbances, or gastrointestinal complaints, can be ascribed to DS itself, leading clinicians to miss the diagnosis for years [66]. Untreated celiac disease not only worsens those problems but can also interfere with medications (for instance, malabsorption of thyroid hormone or anti-diabetic drugs in the gut). Therefore, early introduction of a gluten-free diet in a child with DS can potentially improve not just gastrointestinal health but also stabilize metabolic and endocrine parameters by restoring nutrient uptake and reducing systemic inflammation.

In summary, the gut–endocrine axis in DS is characterized by a complex cross-talk among the intestinal environment, the immune system, and endocrine organs. Triplicated chromosome 21 genes create a pro-inflammatory, autoimmune-prone background, upon which factors like intestinal dysbiosis, recurrent infections, and nutritional deficits further promote a cycle of immune–endocrine disturbance. Clinically, this presents as a high co-occurrence of conditions such as celiac disease, autoimmune thyroiditis, and type 1 diabetes, along with altered gut microbiota and persistent low-grade inflammation. Recognizing these interconnections is crucial for comprehensive management of individuals with DS. For example, treating gut pathology (like celiac or small-intestinal bacterial overgrowth) may alleviate immune stress and improve thyroid or metabolic function, just as optimizing endocrine therapy (e.g., thyroid hormone or insulin) can positively feedback on gastrointestinal well-being.

**Figure 2:**
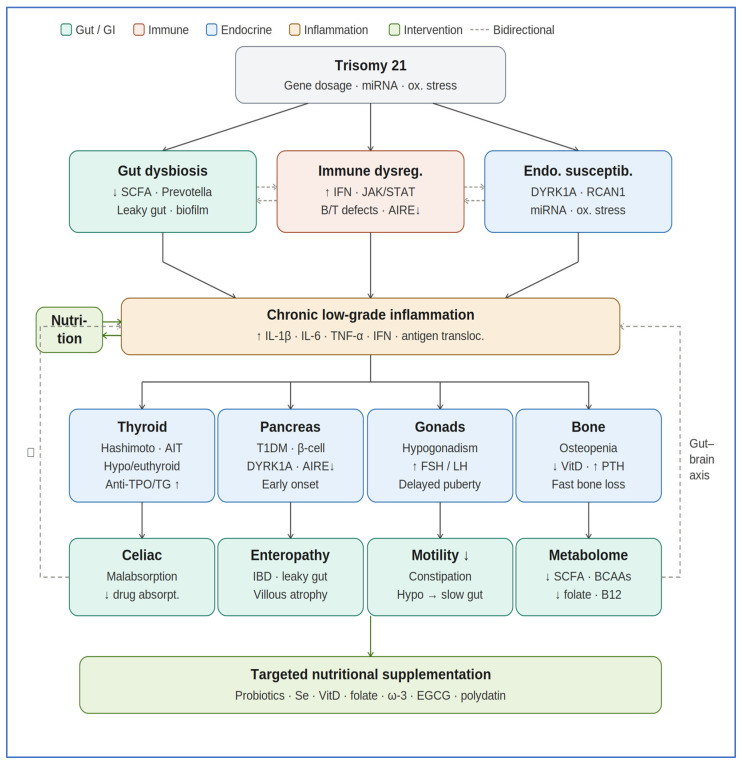
Gut–endocrine axes cross-talk. up-arrow: increase; down-arrow: reduction; side-arrow: to.

A schematic illustration of these bidirectional relationships and key clinical associations in DS is summarized in Figure 2. Ultimately, an integrated, multisystem approach, including vigilant screening and early intervention for both gut and endocrine disorders, is vital to breaking the vicious circle of dysbiosis, autoimmunity, and hormonal imbalance that can occur in DS.

### 3.6. Evidence on Changes Induced by Targeted Nutritional Supplementation

Considering that DS is associated with a persistent low-grade inflammatory state, as described previously, several nutritional and nutraceutical compounds have been investigated for their potential to modulate immune, metabolic, and microbiota-related pathways in DS. It is important to emphasize, however, that the available evidence spans from in vitro and in vivo to small observational studies with a limited number of clinical trials. The level of evidence is, therefore, variable across compounds. Most data derive from non-DS populations or preclinical settings, and robust DS-specific randomized controlled trials are largely lacking. This section reviews the current state of evidence, maintaining a clear distinction between preclinical and observational data, and explicitly evidences where findings cannot be translated into routine clinical recommendations. With this caveat in mind, several nutraceutical compounds have been explored for their potential to target interconnected pathways involved in immune regulation, metabolic homeostasis, and gut microbiota composition.

Among these, probiotics, particularly *Lactobacillus* and *Bifidobacterium* strains, have been associated with improved intestinal barrier integrity and reduced systemic inflammation in the general pediatric population and in inflammatory conditions [9,10]. These effects appear to be mediated through modulation of gut microbiota composition, increased production of short-chain fatty acids, and downregulation of pro-inflammatory pathways [19,20]. However, no randomized controlled trials have been conducted specifically in individuals with DS to date [21,89]. These data are insufficient to support the routine use of probiotics as a therapeutic strategy in this population, and clinical recommendations await dedicated, well-designed DS-specific trials.

Micronutrients also exert a pivotal role in modulating immune and metabolic dysfunctions in DS. Selenium and iodine in particular are key regulators of thyroid function and immune homeostasis. Selenium is required for the activity of seleno-proteins involved in antioxidant defense and redox balance, thereby limiting oxidative stress and regulating inflammatory responses, while iodine is essential for thyroid hormone synthesis and immune regulation [43,93,99,100]. In individuals with DS, deficiencies in these micronutrients have been consistently reported and are associated with increased oxidative stress, impaired thyroid function, and a higher susceptibility to autoimmune thyroiditis. Selenium supplementation (approximately 100 μg/day for about 6 months) has been reported to improve antioxidant capacity and reduce thyroid autoantibody levels in some small studies with considerable methodological heterogeneity, and results are not consistent across studies [1,2,93,100]. These data are insufficient to support routine selenium supplementation in individuals with DS outside the context of a documented deficiency, and no DS-specific clinical guidelines currently endorse this practice as standard care. Similarly, maintaining adequate iodine intake (approximately 90–120 μg/day in children and up to 150 μg/day in adolescents) is essential, as both deficiency and excess may contribute to thyroid dysfunction and autoimmune processes [3]. In addition, vitamin D deficiency, frequently observed in DS, contributes to immune dysregulation and chronic inflammation, and its correction (approximately 400–1000 IU/day, individualized) may improve immune and metabolic profiles [26,66,93,95,99].

Importantly, gut dysbiosis in DS may further exacerbate metabolic and immune alterations by impairing the absorption of key micronutrients, including Iron and Folate, which are essential for hematopoiesis and immune function [19,21,47,101]. Iron deficiency may worsen immune dysregulation, while Folate (vitamin B9) is crucial for DNA synthesis and methylation processes, potentially influencing epigenetic mechanisms that show changes in trisomy 21 [72,93,101]. This highlights a bidirectional relationship between micronutrient status and gut microbiota, where dysbiosis contributes to metabolic imbalance, while micronutrient deficiencies may further affect microbial composition and function.

An important emerging question is whether modulation of gut dysbiosis may reduce the need for micronutrient supplementation. Although restoration of a healthier microbiota could theoretically improve nutrient absorption and reduce systemic inflammation, current evidence in DS is still lacking [19,20,21].

Numerous natural plant-based compounds exhibit protective effects on metabolic changes observed in Down syndrome, such as redox imbalance, reduced mitochondrial function, bioenergetic deficit, oxidative stress, reduced neurogenesis, and neuroplasticity. Among these, polyphenols represent a broad class of molecules with multimodal beneficial effects. In addition to their specific actions, polyphenols are powerful antioxidants that protect cellular structures from oxidative damage caused by free radicals. They also activate sirtuins, regulatory proteins involved in cellular energy metabolism, and stimulate mitochondrial biogenesis, or the synthesis of new mitochondria. Furthermore, polyphenols modulate detoxification enzymes, stimulate the immune system, and reduce neuroinflammation [102].

A randomized, placebo-controlled phase II clinical trial (the TESDAD study) evaluated EGCG, a polyphenol extracted from green tea, administered orally at 9 mg/kg/day in young adults with DS. While EGCG combined with cognitive training showed improvements in some cognitive subdomains in secondary analyses, the trial did not demonstrate significant benefit in primary functional outcomes [103]. Bioavailability data in healthy volunteers informed dosing strategies [104], while safety was addressed in a case report [105]. A small pilot study in young children with DS reported restoration of mitochondrial complex activities in peripheral blood cells [106]. Adequately powered, DS-specific randomized controlled trials are required before any recommendation can be made.

Polydatin is a polyphenol extracted from Polygonum cuspidatum, long used in traditional Asian medicine. Recent studies have demonstrated a wide range of biological effects in humans, including anti-tumor, chemo-sensitizing, cardioprotective, anti-inflammatory, antioxidant, and neuroprotective properties [107,108]. Polydatin has been tested in preclinical studies using cellular and murine models of DS. In the DS mouse model, neonatal supplementation with polydatin proved safe and effective in restoring cognitive and neurodevelopmental deficits, particularly hippocampal neurogenesis [109]. In human fetal fibroblasts with DS, polydatin reactivated mitochondrial bioenergetic activity, reduced excessive ROS production, and prevented DNA damage and cellular senescence induced by oxidative stress [110]. This study evidenced that polydatin was also a modulator of miR-155 [9]. Importantly, all available data on polydatin in DS derive exclusively from preclinical models (cell cultures and rodent models); thus, no clinical recommendations can be made at this stage.

Clinicians should currently exercise caution when interpreting these data, particularly when communicating with patients and families. Micronutrient supplementation (vitamin D, selenium, iodine, zinc) should be based on documented deficiencies rather than on prophylactic use. Table 3 reports micronutrients and nutraceuticals that could potentially have beneficial effects on patients with DS.

## 4. Limitations of Current Evidence and Future Directions

Despite the growing interest in endocrine and gastrointestinal disorders in DS, several limitations still affect the current body of evidence. First, a marked heterogeneity exists across available studies in terms of patient age, clinical phenotypes, diagnostic criteria, study design, and sample size. Many reports combine pediatric and adult populations or include individuals with highly variable comorbidities, making direct comparisons difficult and limiting the generalizability of findings. In addition, differences in nutritional status, pharmacological treatments, geographic background, and environmental exposures may further contribute to variability in reported outcomes.

Second, longitudinal pediatric studies are very limited. Most available evidence derives from cross-sectional analyses or retrospective cohorts, which provide only a partial understanding of disease evolution over time. Consequently, the temporal relationship between immune dysregulation, endocrine dysfunction, gastrointestinal manifestations, and metabolic alterations in children with DS remains incompletely characterized. Long-term prospective studies are, therefore, needed to better define the natural history of these conditions, identify early predictive biomarkers, and evaluate the impact of preventive and therapeutic interventions throughout development.

Finally, although increasing attention has been devoted to the gut microbiome in DS, current evidence remains limited and largely exploratory. Most microbiome studies include relatively small cohorts, lack standardized methodologies, and often rely on heterogeneous sequencing and bioinformatic approaches, reducing reproducibility across studies. Moreover, pediatric data are particularly scarce, and little is currently known about the longitudinal evolution of gut microbial composition during childhood and adolescence in DS. Whether dysbiosis represents a primary contributor to immune and endocrine dysfunction or rather a secondary consequence of chronic inflammation and comorbidities remains unclear. Future multicenter studies integrating microbiome, metabolomic, immunologic, and clinical data will be essential to clarify the mechanistic role of the gut–immune–endocrine axis in DS and to assess the potential clinical utility of microbiota-targeted interventions.

## 5. Conclusions

DS is a complex multisystem condition in which trisomy 21 exerts widespread biological effects extending far beyond neurodevelopmental impairment. This review highlights that endocrine and gastrointestinal disorders in DS should not be considered isolated comorbidities, but rather interconnected manifestations of a shared pathophysiological framework characterized by gene dosage imbalance, immune dysregulation, chronic low-grade inflammation, epigenetic alterations, metabolic disturbances, and gut microbiota dysbiosis.

Endocrine abnormalities—including thyroid dysfunction, type 1 diabetes mellitus, growth impairment, obesity, altered pubertal development, and reduced bone mineral density—occur more frequently and often present earlier or atypically compared with the general population. In parallel, gastrointestinal conditions, ranging from congenital anomalies to functional disorders, and autoimmune enteropathies such as celiac disease, significantly contribute to morbidity and may further exacerbate systemic inflammation and nutritional deficiencies.

Increasing evidence supports the existence of a tightly interconnected gut–endocrine–immune axis in DS. Alterations in gut microbiota composition, increased intestinal permeability, and disrupted microbial metabolite profiles appear to interact bidirectionally with the immune and endocrine pathways, fostering a pro-inflammatory and autoimmune-prone environment. This integrated dysfunction likely explains the frequent clustering of autoimmune conditions, including thyroiditis, celiac disease, and type 1 diabetes, within the same individual.

Epigenetic mechanisms, particularly chromosome 21-encoded microRNAs, further amplify these processes by modulating immune responses, metabolic regulation, and tissue homeostasis. Additionally, micronutrient imbalances, oxidative stress, and mitochondrial dysfunction contribute to the heterogeneity and progression of clinical manifestations.

From a clinical perspective, these findings underscore the importance of a comprehensive and multidisciplinary approach to DS management. Lifelong surveillance, early recognition of atypical presentations, and coordinated care strategies addressing endocrine, gastrointestinal, immune, and nutritional aspects are essential to improve health outcomes. Nutritional and microbiota-targeted interventions have been explored as potential adjunctive strategies; however, for most of the compounds reviewed in Section 3.6, the current evidence is predominantly preclinical, observational, or derived from non-DS populations, and is insufficient to support routine clinical use. Clinicians and families should be clearly informed that nutraceuticals such as probiotics (beyond general pediatric indications), EGCG, and Polydatin, as well as micronutrient supplementation beyond the correction of documented deficiencies, are not recommended as standard-of-care interventions in DS. Well-designed, adequately powered randomized controlled trials specifically targeting individuals with DS are urgently needed before any such recommendations can be established.

Future research should prioritize longitudinal and integrative studies combining immunological, microbiome, and metabolic profiling to better elucidate causal mechanisms within the gut–endocrine axis. Such efforts may enable the identification of high-risk individuals and support the development of personalized preventive and therapeutic approaches. Advancing our understanding of these interconnected systems holds the potential not only to enhance quality of life in individuals with DS but also to provide broader insights into the mechanisms underlying autoimmunity and chronic multisystem diseases.

## Figures and Tables

**Table 1:** Prevalence of the most frequent autoimmune conditions in individuals with Down Syndrome compared with the general population (adapted from Hom B et al. [19]).

Clinical Category	Condition	Prevalence in Individuals with Down Syndrome	Prevalence in the General Population
Endocrine disorders	Hashimoto’s thyroiditis	13–34%	~2–3%
	Graves’ disease	0.66%	~0.02%
	Type 1 diabetes mellitus	1.7%	~0.4%
Gastrointestinal disorders	Celiac disease	5.8%	0.5–2%

**Table 2:** Gastrointestinal and Endocrine Conditions in DS: Prevalence, Pathophysiological Interactions, Clinical Implications, and Management. Abbreviations: DS (Down syndrome).

Condition	Prevalence in DS vs. General Population	Key Gut–Endocrine Link	Clinical Impact and Management
Autoimmune Thyroiditis (Hashimoto’s)	13–34% (vs. 1–2%)	Low thyroid hormones reduce gut motility, worsening constipation	Requires annual screening; clinical signs (lethargy, constipation) often overlap with DS phenotype
Celiac Disease	5–7% (vs. ~1%)	Malabsorption of thyroid hormone or anti-diabetic medications; chronic inflammation originating in the gut	Universal screening by age 2–3; gluten-free diet can stabilize metabolic parameters and nutrient uptake
Type 1 Diabetes (T1DM)	1–4% (vs. ~1% in pediatrics)	Hypothyroidism can deteriorate glycemic control	Integrated approach: thyroid replacement can help stabilize blood glucose
Intestinal Dysbiosis	High (distinctive profile)	Enrichment of pro-inflammatory bacteria (*Prevotella*, *Escherichia*/*Shigella*) correlates with systemic cytokines and impairs nutrient/medication absorption and metabolism	Diet optimization and cautious consideration of microbiota-targeted strategies, such as probiotics
Nutritional Deficiencies (e.g., Vitamin D)	Common; no standard % consistently reported	Deficiency (often due to poor diet/sedentary lifestyle) skews immune homeostasis and thyroid function	Adequate micronutrient levels are essential for hormone production and immune regulation; supplementation often necessary

**Table 3:** Targeted micronutrients and nutraceuticals that can potentially have beneficial effects on patients with Down syndrome. Abbreviations: AIT (autoimmune thyroiditis), Abs (antibodies), SePP (selenoprotein P), GP (selenocysteine-containing glutathione peroxidase 3), anti-TPO (anti-thyroid peroxidase), anti-TG (anti-tireoglobulin), DS (Down syndrome), EGCG (epigallocatechin gallate), FAs (fatty acids), EPA (eicosapentaenoic acid), DHA (docosahexaenoic acid), PLD (polydatin).

Micronutrients and Nutraceuticals	Recommendations and Suggested Dose (If Studied)	Function and Effects of Supplementation	References
**Selenium**	Selenium-rich diet. Supplementation: ≈100 mcg/day (≈6 months) in studies involving children with AIT.	Deficiency increased thyroid autoimmunity. Supplementation is associated with - anti-TPO and anti-TG Ab reduction; - improved thyroid function; - improved antioxidant status.	[111,112]
**Iodine**	Adequate dietary intake: RDA ≈ 90–150 µg/day depending on age (standard pediatric recommendations).	- Essential for thyroid hormone synthesis. - Both deficiency and excess iodine can worsen thyroid dysfunction.	[113]
**Zinc**	Supplementation if deficient or poor dietary intake. No DS-specific dose recommended. Studied regimens include: ≈1 mg/kg/day (3–6 months); ≈30 mg/day (4 weeks—adolescents).	- Improved antioxidant status. - Improved thyroid function.	[114,115,116]
**Vitamin D**	Routine monitoring recommended. Supplementation often required. Individualization needed: ~400–1000 IU/day (higher in DS patients with obesity and/or autoimmune disease or if deficient).	- Improved bone health markers. - Enhanced immune function. - Supported thyroid function. - No significant cognitive benefit.	[66]
**Folic acid and vitamin B12**	Evaluate nutritional status. Supplementation if deficient according to general pediatric guidelines.	- Restored Folate stores, normalizes homocysteine levels and vit. B12 levels. - Limited evidence on cognitive benefit.	[117]
**Omega-3 Fatty Acids**	200–500 mg/day (for 3–6 months) (general pediatric population).	- Anti-inflammatory and immunomodulatory effect; - Supports brain development; - Normalizes mitochondria respiratory chain complex activities. - A negative association has been reported between Omega-3 FA levels and autoimmune thyroiditis. - Additional research needed to confirm benefit in DS.	[108,118]
**Probiotics (*Lactobacillus*, *Bifidobacterium*)**	10^9^–10^10^ CFU daily (general pediatric population). Ongoing studies in DS population.	- Reduced dysbiosis-driven gut and systemic inflammation. - Improved bowel motility. - Possible improvements in metabolic and cognitive markers. - No DS-specific studies on glycemic control or thyroid function.	[21,94,119]
**EGCG—*Epigallocatechin Gallate*** (green tea extract)	EGCG: 10 mg/kg/day, suspended in 250–500 mg EPA + DHA/day (for 6 months). 0.5% EGCG: 10 mg/kg/day (max 400 mg/day) (for 6 months). Monitor possible decline of plasma Folate. EGCG: 10 mg/kg/day, suspended in 250–500 mg/day Omega-3 FAs EPA + DHA/day (for 6 months) (studied in DS children).	- Normalizes mitochondria respiratory chain complex activities. - No significant improvement in cognitive outcomes. - Possible improvements in executive function tasks, working memory, planning, and academic skills.	[103,108,120,121]
**Polydatin**	20 μM PLD concentration (human fibroblasts with trisomy 21 cell cultures). 2.5 mg/kg dose (DS mouse model). 20–80 mg/day (safe dose in human studies, not DS-specific).	- Restored mitochondrial energy metabolism, reduces oxidative stress, prevents premature cellular aging, regulates miR-155 signaling, enhances mitophagy. - Early postnatal treatment with Polydatin restores neurogenesis, neuronal structure, and cognitive function.	[107,109,110,122,123]
**Coenzyme Q10**	High-dose coenzyme Q10: 10 mg/kg/day (2–3 months).	- Well tolerated; effective in improving mitochondrial function and reducing pro-inflammatory state. - Further studies needed to determine whether correction of oxidative imbalance improves clinical outcomes.	[124,125,126]

## Data Availability

No new data were created or analyzed in this study. Data sharing is not applicable to this article.

## Abbreviations

The following abbreviations are used in this manuscript:

DS	Down Syndrome
CD	Celiac Disease
T1DM	Type 1 Diabetes
IBDs	Inflammatory bowel diseases
miRNAs	microRNAs
